# HSPB1 alleviates acute-on-chronic liver failure via the P53/Bax pathway

**DOI:** 10.1515/biol-2022-0919

**Published:** 2024-07-24

**Authors:** Zhixiang Zhang, Jinwei Guo, Jincan Zhu

**Affiliations:** Department of Infectious Diseases, Shenzhen Guangming District People’s Hospital, Shenzhen, Guangdong, 518106, China

**Keywords:** acute-on-chronic liver failure, HSPB1, P53, Bax, LPS

## Abstract

The mortality rate of acute-on-chronic liver failure (ACLF) remains significantly elevated; hence, this study aimed to investigate the impact of heat shock protein family B (small) member 1 (HSPB1) on ACLF *in vivo* and *in vitro* and the underlying mechanism. This study used the ACLF mouse model, and liver damage extent was studied employing Masson trichrome, hematoxylin and eosin (H&E), Sirius red staining, and serum biochemical indices. Similarly, hepatocyte injury in lipopolysaccharide (LPS)-induced L02 cells was evaluated using cell counting kit-8 assay, enzymatic activity, flow cytometry, and TUNEL assay, while the underlying mechanism was investigated using western blot. Results showed that the morphology of liver tissue in ACLF mice was changed and was characterized by cirrhosis, fibrosis, collagen fiber deposition, inflammatory cell infiltration, and elevated liver injury indices. Moreover, HSPB1 was upregulated in both ACLF patients and mice, where overexpressing HSPB1 was found to inhibit ACLF-induced liver damage. Similarly, the HSPB1 expression in LPS-treated L02 cell lines was also increased, where overexpressing HSPB1 was found to promote cell viability, inhibit liver injury-related enzyme activity, and suppress apoptosis. Mechanistic investigations revealed that HSPB1 was responsible for inhibiting p-P53 and Bax protein levels, where activated P53 counteracted HSPB1’s effects on cellular behaviors. In conclusion, HSPB1 attenuated ACLF-induced liver injury *in vivo* and inhibited LPS-induced hepatocyte damage *in vitro*, suggesting that HSPB1 may be a novel target for ACLF therapy.

## 1 Introduction

Acute-on-chronic liver failure (ACLF) is a clinical indication of acute liver decompensation based on chronic hepatopathy, typically characterized by systemic inflammation, extrahepatic organ injury, and a poor short-term prognosis [1], with significantly high global incidence and mortality [2]. The 28-day mortality in China due to ACLF has been reported to be close to 50% [3]. The primary causes of ACLF have been reported to be infections, alcohol consumption, hepatitis, and immunosuppression, and it can proceed to various complications, including bleeding, rehydration, hepatic encephalopathy, and secondary infection [4], with unavailability of specific ACLF therapeutic options. Liver transplantation has been regarded as the gold standard for treating ACLF, but it suffers limitations such as insufficient donors and challenging intensive care management of patients [5]. Therefore, it is imperative to understand ACLF pathogenesis to propose a novel treatment strategy.

Heat shock protein family B (small) member 1 (HSPB1), which belongs to the small heat shock protein, is upregulated when cells go into heat shock [6], where its constitutive expression increases resistance to stress and injury under pathological conditions [7]. HSPB1 has been reported to regulate cellular activity in response to stimulation due to its rapid phosphorylation [8]. Multiple biological functions have been reported for HSPB1, including cellular aging, by acting as a molecular chaperone to protect partially folded or unfolded proteins during stress in humans [9]. Moreover, it has also been found to exert a potent neuroprotective effect against brain injury [10], possesses anti-cancer effects by regulating ferroptosis [11], and has a prognosis value for cancers [11]. Similarly, elevated HSPB1 has recently been found to improve pathological changes in the brain during hepatic encephalopathy [12]. However, the role of HSPB1 in ACLF remains unexplored.

Therefore, this study aimed to investigate the effects of HSPB1 in ACLF animal and cell models and explore the underlying mechanisms. It was hypothesized that HSPB1 affects liver function both *in vivo* and *in vitro*, and the data are envisaged to provide a novel approach for treating ACLF.

## 2 Materials and methods

### 2.1 ACLF mice modeling

The animal protocol was approved by the Ethics Committee of Shenzhen Guangming District People’s Hospital. C57BL/6 J mice (male, 20–22 g) were housed at 22 ± 2°C, 12/12 h light/dark, 50–60% humidity. All mice were randomly divided into four groups: sham, ACLF model, model + negative control lentivirus (Lv-NC), and model + HSPB1 overexpression lentivirus (Lv-HSPB1), with six mice in each group. To establish the ACLF model, carbon tetrachloride (CCl4) was diluted in olive oil in a ratio of 1:10, and the mice were intraperitoneally injected with 0.2 ml/kg of CCl4 for 8 weeks, three times a week. Twenty-four hours after the last injection of CCl4, the mice in the model group were intraperitoneally injected with 320 mg/kg d-galactosamine (d-Gal) and 50 μg/kg lipopolysaccharide (LPS) (d-Gal and LPS were dissolved in normal saline) to cause acute injury. The mice in the sham group were injected with the same dose of normal saline at the same time. To overexpress HSPB1 in ACLF mice, Lv-HSPB1 and the control Lv-NC were purchased from Genepharma (China). During the 8th week of CCl4 infection, the mice were intravenously injected through the tail with 0.5 ml of 5 × 10^8^ PFU/ml Lv-HSPB1 and Lv-NC twice a week. After d-Gal/LPS was injected for 6 h, serum was collected from all mice. Then, all mice were sacrificed, and liver tissues were obtained.


**Ethical approval:** The research related to animal use has been complied with all the relevant national regulations and institutional policies for the care and use of animals and has been approved by the Ethics Committee of Shenzhen Guangming District People’s Hospital.

### 2.2 H&E staining assay

Liver tissue was immobilized overnight in formalin and prepared into paraffin sections at 5-μm-thick. Paraffin sections were dewaxed and rehydrated. Then, the sections were stained with hematoxylin for 5 min, followed by stained with eosin for 1 min. The sections were visualized using a microscope (Leica, Germany).

### 2.3 Masson staining assay

Paraffin sections were dewaxed and rehydrated. The sections were stained using dyes from a Masson trichrome staining kit (Sbjbio, China) according to the manufacturer’s protocol. The results were visualized using a microscope. Masson stained area was quantified using Image J software.

### 2.4 Sirius red staining assay

Paraffin sections were dewaxed and rehydrated. The sections were stained with celestine blue dye for 10 min. Following washing with distilled water, the sections were stained with Sirius red for 1 h, then dehydrated and permeabilized. The sections were visualized using a microscope. Sirius red area was quantified using Image J software.

### 2.5 Detection of blood biochemical indices

Aspartate aminotransferase (AST), alanine aminotransferase (ALT), and total bilirubin (Tbil) levels and the percentage of neutrophils in the serum of mice were examined using an automatic biochemical analyzer (Beckman Coulter, USA).

### 2.6 Clinical samples

A total of 38 patients with ACLF participated in this study. Meantime, 38 patients with drug-induced liver injury were served as non-ACLF. The serum sample was collected from each subject. This study was approved by the Ethics Committee of Shenzhen Guangming District People’s Hospital. Written informed consent was obtained from all subjects.


**Informed consent:** Informed consent has been obtained from all individuals included in this study.
**Ethical approval:** The research related to human use has been complied with all the relevant national regulations, institutional policies and in accordance with the tenets of the Helsinki Declaration, and has been approved by the authors’ institutional review board or equivalent committee.

### 2.7 Cell culture

Human normal liver cells (L02) purchased from ATCC (USA) were cultured in RPMI-1640 containing 10% fetal bovine serum (Hakata, Japan). All cells were incubated at 37°C with 5% CO_2_.

### 2.8 Cell transfection

HSPB1 overexpression vector and empty vector were purchased from Genepharma. L02 cells were seeded into six-well plates at the density of 2 × 10^5^ cells/well and transfected with these vectors using Lipofectamine 3000 (Invitrogen, USA). After 48 h, transfection efficiency was examined using quantitative real-time PCR (qRT-PCR).

### 2.9 Cell treatment

LPS (10.0 µg/ml) was used to treat L02 cells at 37°C for 24 h to induce hepatocyte damage. To activate P53, CBL0137 (150 nM; APExBIO, USA) was used to treat L02 cells for 24 h.

### 2.10 qRT-PCR

Total RNA was isolated from the serum and cells using Trizol reagent (Invitrogen). The first chain of cDNA was synthesized using the Golden 1st cDNA Synthesis Kit (HaiGene, China). A reverse transcription product was used to perform qPCR using RAPA3G SYBR Green qPCR Mix (HaiGene, China). The reaction conditions were 95°C for 5 min, followed by 40 cycles of 95°C for 10 s and 60°C for 20 s. The reaction instrument was a CFX96 Real-Time PCR system (Bio-Rad, USA). The expression of HSPB1 was calculated using the 2^−ΔΔCt^ method, with GAPDH as the internal reference. The primers (5′–>3′) were used as follows: HSPB1 sense, CTCTGAAGGGTCCGAAGTGAT; anti-sense, ATTCCTGTGGTGGTCCAAAAC; GAPDH sense, GGAGCGAGATCCCTCCAAAAT; anti-sense, GGCTGTTGTCATACTTCTCATGG.

### 2.11 Cell Counting Kit-8 (CCK-8) assay

Cell viability was evaluated using a CCK-8 kit (Beyotime, China). Transfected L02 cells (2 × 10^3^ cells) were seeded into 96-well plates and incubated with LPS for 24 h with or without CBL0137 treatment for 24 h. Afterward, a CCK-8 solution (10 µl) was added. After 4 h, the absorbance value was measured using a microplate reader at 450 nm (Bio-Rad, USA).

### 2.12 Detection of AST, ALT, and lactate dehydrogenase (LDH) activities

After LPS or CBL0137 treatment, AST activity was analyzed using AST or SGOT Activity Assay Kit (Biovision, USA); ALT activity was measured using ALT or SGPT Activity Assay Kit (Biovision); and LDH activity was detected using LDH Activity Assay Kit (Solabio, China) following the manufacturer’s protocol.

### 2.13 Flow cytometry

Cell apoptosis was evaluated using Annexin V-FITC/7AAD apoptosis detection kit (Procell, China). Briefly, cells (2 × 10^5^ cells) were suspended in 500 μl of Annexin V binding buffer. Then, the cell suspension was incubated with Annexin V-FITC (5 μl) and 7-AAD solution (5 μl) for 15 min in the dark. Flow cytometry was conducted within 1 h.

### 2.14 TUNEL assay

After washing with PBS, the cells were fixed with 4% paraformaldehyde for 0.5 h, followed by permeabilized with 0.3% Triton X-100 for 5 min. Following blocking using 0.3% H_2_O_2_ for 20 min, the cells were incubated with TUNEL solution (50 μl) for 1 h at 37°C. The stained cells were visualized using a fluorescence microscope. DAPI was the reference for staining the nucleus.

### 2.15 Western blot

Following treatment, after washing with PBS, the cells were lysed using radioimmunoprecipitation assay lysis buffer on the ice. Protein concentration was tested by the BCA Protein Assay Kit (HaiGene). The lysate was run on the 10% SDS-PAGE for separation. Blocking was determined using 5% skim milk for 1 h. The membranes were incubated with primary antibodies at 4°C overnight and incubated with secondary antibody at 25°C for 2 h. The immune complexes were visualized by ultra-sensitive electrochemiluminescence solution (HaiGene). β-Actin was the internal reference. Gray analysis was assessed using Image J software. The antibodies (Abcam, USA) used here were as follows: anti-p53 (ab26, 1:500), anti-phosphorylated (p)-p53 (ab122898, 1:1,000), anti-Bax (ab3191, 1:250), anti-β-actin (ab8226, 1:1,000), and rabbit anti-mouse IgG (ab6728, 1:5,000).

### 2.16 Statistical analysis

All data are shown as mean ± SD. The differences among multiple groups or between two groups were analyzed by one-way ANOVA or Student’s *t*-test using GraphPad Prism 7 software. *P* < 0.05 is considered to be a significant difference.

## 3 Results

### 3.1 Overexpression of HSPB1 alleviated liver failure in ACLF mice

**Figure 1:**
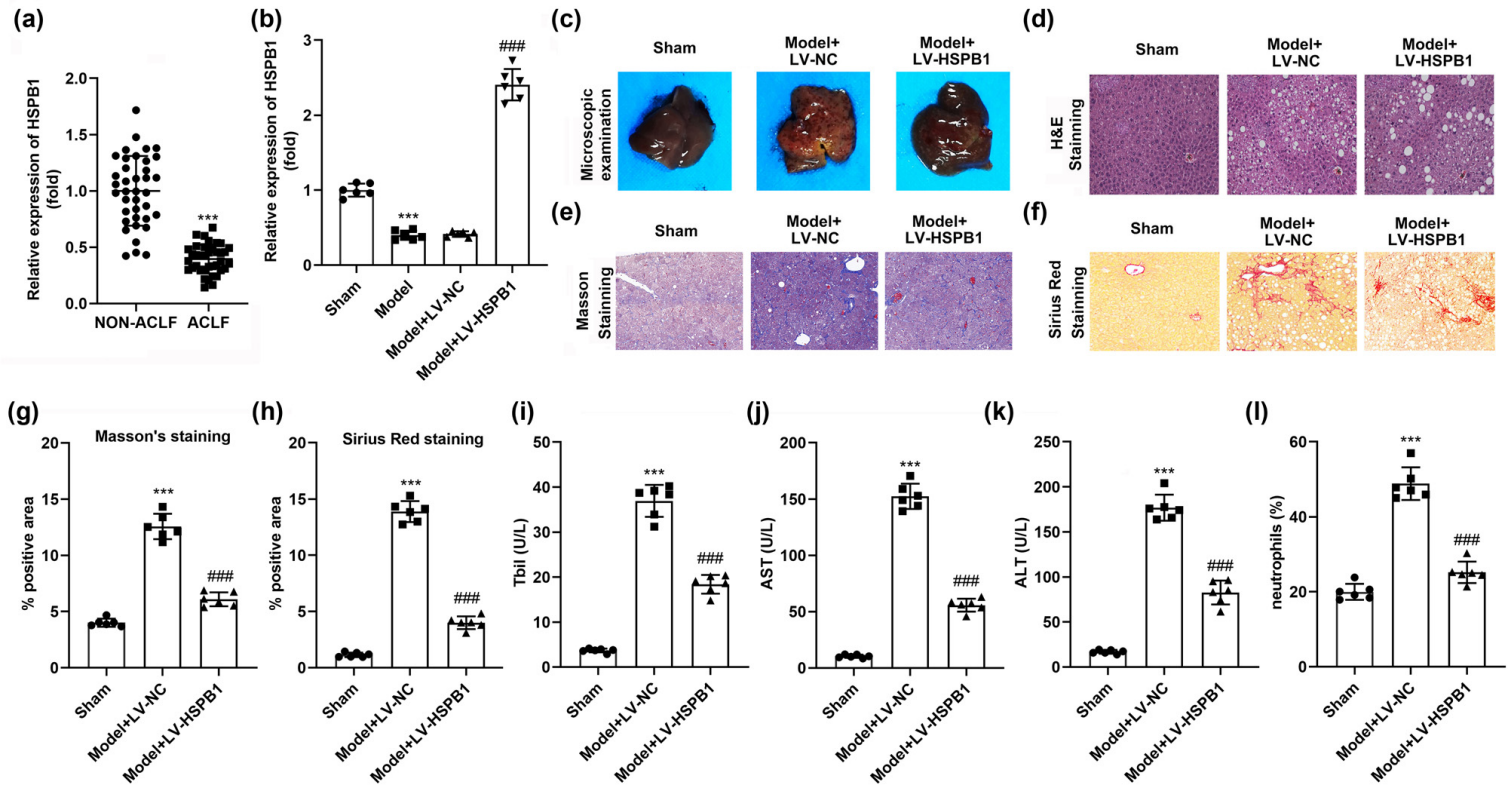
Overexpression of HSPB1 alleviated liver failure in the ACLF mice model. (a) HSPB1 expression in patients with or without ACLF. (b) HSPB1 expression in ACLF mice and LV-HSPB1 transfected model. (c) The liver samples were collected after the mice were sacrificed. Liver tissues were stained using (d) H&E, (e) Masson, and (f) Sirius red dyes. (g) Quantification of (d). (h) Quantification of (f). (i) Tbil, (j) AST, (k) ALT levels, and (l) the percentage of neutrophils in the serum of mice were detected. ***P < 0.001. ^###^ *P* < 0.001.

The *in vivo* levels of HSPB1 were found to be downregulated in ACLF patients compared to the non-ACLF group, as shown in Figure 1a. Similarly, they were found to be lowly expressed in ACLF mice while upregulated with LV-HSPB1 (Figure 1b). Multiple hepatic pathological changes including cirrhosis and fibrosis were observed in the ACLF model, which was found to be alleviated by HSPB1 (Figure 1c). H&E results indicated significant hepatic injury in the ACLF mouse model, including irregular cellular arrangement, necrotic areas, and inflammatory cell infiltration, which were improved following HSPB1 overexpression, cementing the HSPB1 role in inhibiting liver damage (Figure 1d). Masson trichrome histological data suggested that HSPB1 inhibited ACLF-induced collagen fiber deposition and inflammatory factors infiltration (Figure 1e and g). Similarly, Sirius red staining data showed that HSPB1 induced collagen fiber deposition in ACLF mice (Figure 1f and h). Moreover, serum indicators, including Tbil, AST, ALT levels, and neutrophil percentage, were elevated in ACLF mice, which were reduced with overexpressing HSPB1 (Figure 1i–l).

### 3.2 Overexpression of HSPB1 ameliorates LPS-induced liver cell dysfunction

**Figure 2:**
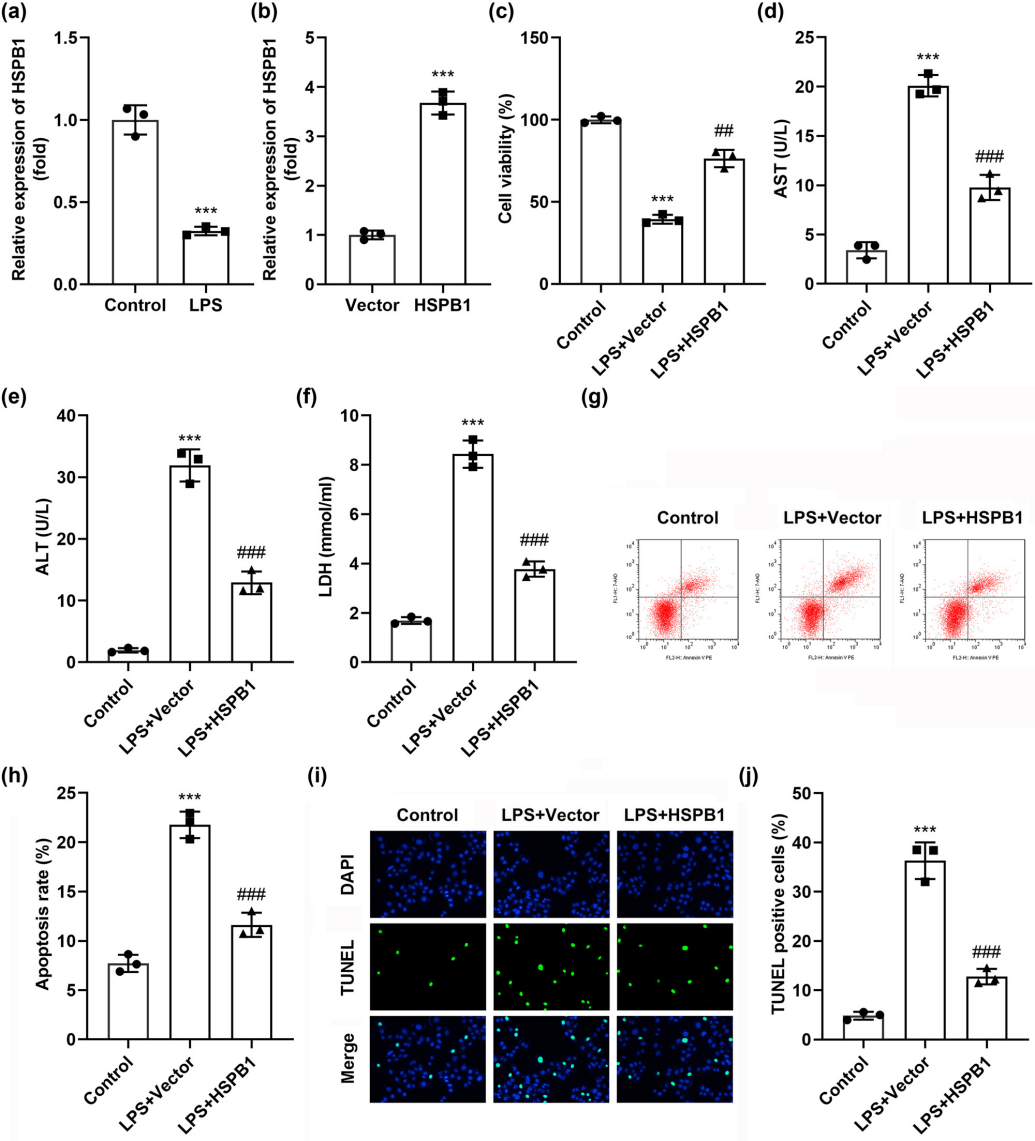
Overexpression of HSPB1 ameliorates LPS-induced liver cell dysfunction. (a) The expression of HSPB1 in LPS-treated cells. (b) HSPB1 expression after transfection with HSPB1 overexpression vector. (c) CCK-8 assay analyzed cell viability. (d) AST, (e) ALT, and (f) LDH activities. (g) and (h) Flow cytometry evaluated cell apoptosis. (i, j) Cell apoptosis was evaluated by TUNEL assay. ***P < 0.001. ^###^ *P* < 0.001. ^##^ *P* < 0.01.

The HSPB1 level was found to be significantly downregulated post-LPS treatment *in vitro* (Figure 2a). The HSPB1 overexpressing vector was then transfected, which translated into significantly elevated HSPB1 expression (Figure 2b). Moreover, LPS treatment resulted in the inhibition of cell viability, which was markedly promoted by HSPB1, as shown in Figure 2c, in addition to significantly increased AST, ALT, and LDH activities, which were attenuated by overexpressing HSPB1 (Figure 2d–f). Flow cytometry and TUNNEL assay results showed that LPS treatment also facilitated cellular apoptosis, which was rescued by HSPB1 (Figure 2g–j).

### 3.3 HSPB1 inactivates the P53/Bax pathway *in vivo* and *in vitro*

**Figure 3:**
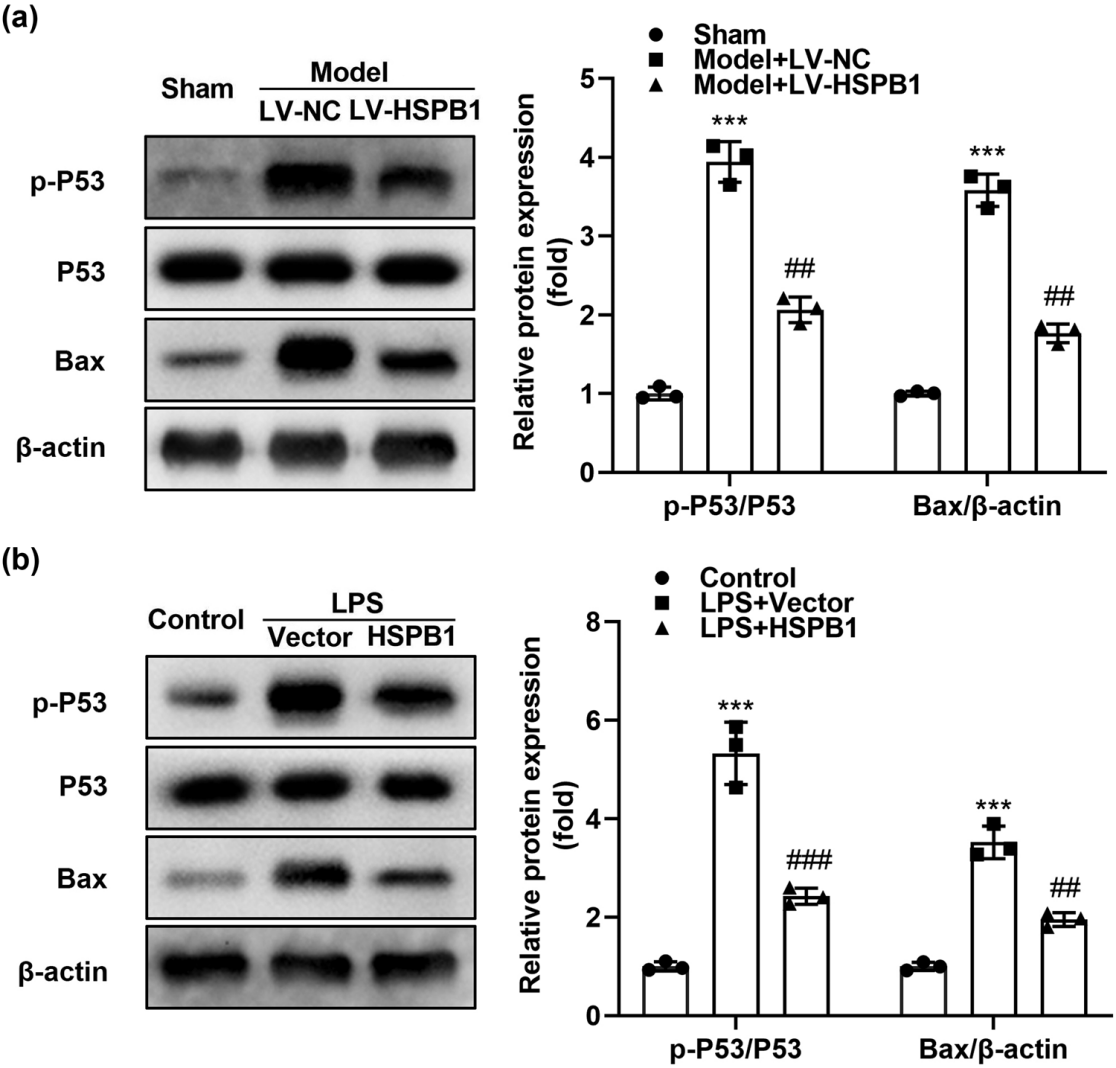
HSPB1 suppresses the activation of the P53/Bax pathway. (a) p-P53, P53, and Bax protein levels were detected and quantified in the liver tissues of mice. (b) p-P53, P53, and Bax protein levels were detected in L02 cells and quantified. ***P < 0.001. ^###^ *P* < 0.001. ^##^ *P* < 0.01.

The HSPB1 mechanism in alleviating hepatic injury was evaluated by examining p-P53, P53, and Bax protein levels. Results showed that the levels of p-P53 and Bax were significantly elevated in ACLF mice and LPS-treated L02 cells, which were reversed by overexpressing HSPB1; however, the expression of P53 in all groups was insignificantly different (Figure 3a and b).

### 3.4 HSPB1 alleviates LPS-induced hepatocyte injury via the P53/Bax pathway

**Figure 4:**
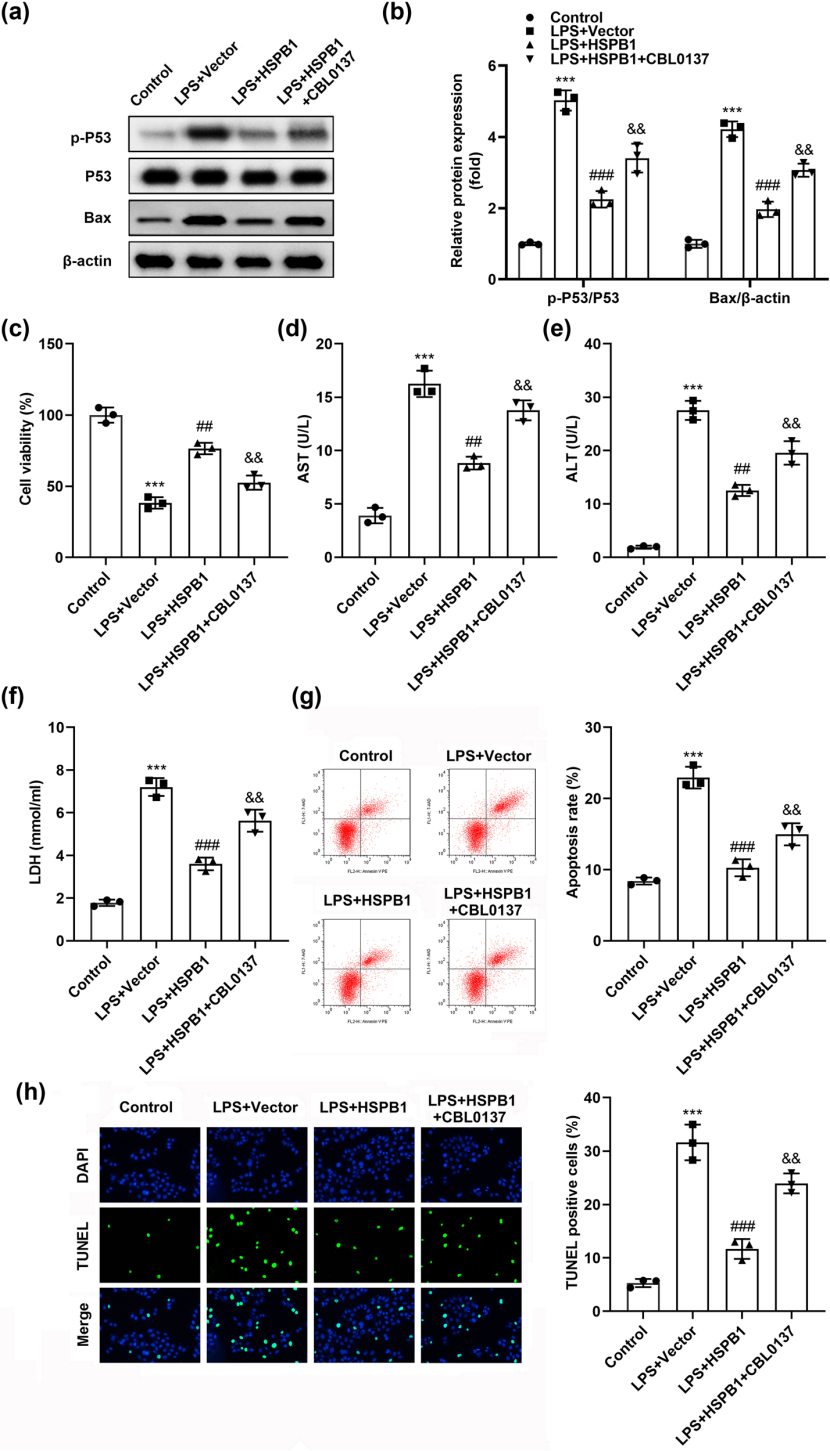
HSPB1 alleviates LPS-induced hepatocyte injury via the P53/Bax pathway. (a) and (b) The p-P53, P53, and Bax protein levels were tested in L02 cells and quantified. (c) CCK-8 assay evaluated cell viability. The activities of (d) AST, (e) ALT, and (f) LDH were assessed. (g) Flow cytometry and (h) TUNEL assay were conducted to analyze apoptosis. ***P < 0.001. ^###^ *P* < 0.001. ^##^ *P* < 0.01. ^&&^ *P* < 0.01.

Subsequently, P53/Bax pathways were activated by treating cells with CBL0137, which significantly reversed the reduction of P53 and Bax’s levels induced by HSPB1 in LPS-treated cells (Figure 4a and b), in addition to counteracting HSPB1-induced promotion of cell viability (Figure 4c). Similarly, HSPB1-induced inhibition of AST, ALT, and LDH activities in LPS-treated cells was rescued by CBL0137 treatment (Figure 4d–f), in addition to reversal of cellular apoptosis suppression induced by HSPB1 in LPS-treated cells (Figure 4g and h).

## 4 Discussion

ACLF is characterized by acute compensatory decompensation, organ failure, and a high short-term mortality rate. The underlying diseases of ACLF include cholestasis, metabolic liver disease, chronic hepatitis, and non-alcoholic or alcoholic steatohepatitis [13]. Patients with chronic cirrhosis and liver fibrosis are prone to developing ACLF, where inflammatory cell infiltration is regarded as the primary cause of tissue and organ damage [14]. Patients with cirrhosis typically exhibit neutropenia, whereas patients with ACLF demonstrate elevated neutrophil counts [15]. Similarly, the classical markers of liver damage (ALT, AST, and Tbil) are reported to be significantly elevated in ACLF mouse and rat models [16, 17]. Our results indicated an altered hepatic morphology in the ACLF mice model, accompanied by elevated Tbil, ALT, AST, and neutrophils, cementing successful ACLF model establishment, where ACLF was responsible for damaged liver in mice.

HSPB1 is associated with multiple diseases, such as atherosclerosis, inflammation, neuropathy, and malignancy, via regulating cell apoptosis, proliferation, autophagy, and metastasis [18–21]. HSPB1 has previously been found to exert hepatoprotective effects, such as HSPB1 being expressed in liver cells and inhibiting inflammatory injury [22]. Moreover, HSPB1 has also been found to exert an anti-viral effect through downstream anti-viral effector proteins during hepatitis B infection [23]. Conversely, high HSPB1 expressions have also been found to be associated with liver injury; its expressions were elevated in patients with liver cirrhosis, where knocking down HSPB1 alleviated fibrosis [24]. Similarly, higher HSPB1 expression was also found to be associated with NF-κB pathway-mediated liver inflammation responses [25]. However, whether HSPB1 promotes or inhibits liver damage in ACLF is unknown. Our results showed that HSPB1 was downregulated in ACLF patients and mice, where overexpressing HSPB1 alleviated ACLF-induced liver injury, suggesting HSPB1 could potentially improve liver function injury induced by ACLF. Then, we used an *in vitro* study to investigate how HSPB1 alleviated liver damage. The ACLF pathogenesis involves multiple cell death mechanisms such as apoptosis, pyroptosis, and necroptosis [26]. Here, we focused on the apoptosis of hepatocytes. Our data showed that overexpressing HSPB1 promoted cell viability and inhibited hepatocellular injury and apoptosis, suggesting that HSPB1 attenuated liver damage by suppressing liver cell apoptosis.

The molecular mechanisms of HSPB1 action via apoptosis regulators were studied based on the vital role of apoptosis in ACLF progression. P53 possesses the ability to induce cell apoptosis. Previous studies have revealed that P53-mediated apoptosis is involved in ACLF. For example, liver pathological damage was alleviated by suppressing liver cell apoptosis in ACLF rats through apoptosis pathways dependent or independent of P53 [27]. Similarly, San Huang Yin Chi decoction has also been found to prevent and treat ACLF by regulating the P53 apoptosis signaling pathway [28]. A pro-apoptotic gene, Bax, is a downstream factor of P53 that can be upregulated by activated P53 to trigger cell apoptosis [29]. The P53/Bax pathway was reported to be associated with numerous diseases, including cancers, acute hepatic injury, and neuronal injury [30–32], where Bax has been regarded as a key factor in facilitating cell apoptosis in ACLF [33, 34]. Hence, we analyzed the role of the P53/Bax axis in ACLF, where p-P53 and Bax were found to be upregulated in ACLF and LPS-treated cells, which HSPB1 hampered. Similarly, activation of the P53/Bax axis was found to reverse the protective effect of HSPB1 on liver cell injury and apoptosis, suggesting HSPB1 ameliorated liver cell damage, at least partly by inactivating the P53/Bax pathway. However, the relationship between P53 and Bax in ACLF remains unclear, in addition to how HSPB1 regulates P53 and Bax, which were the major limitations of this study and will be investigated in the future.

In conclusion, this study showed that HSPB1 expression was low in ACLF patients, mice, and LPS-induced L02 cells. HSPB1 overexpression partially prevented hepatocyte damage and apoptosis by deactivating the P53/Bax pathway, potentially reducing liver injury caused by ACLF, making HSPB1 a novel target for treating ACLF.
